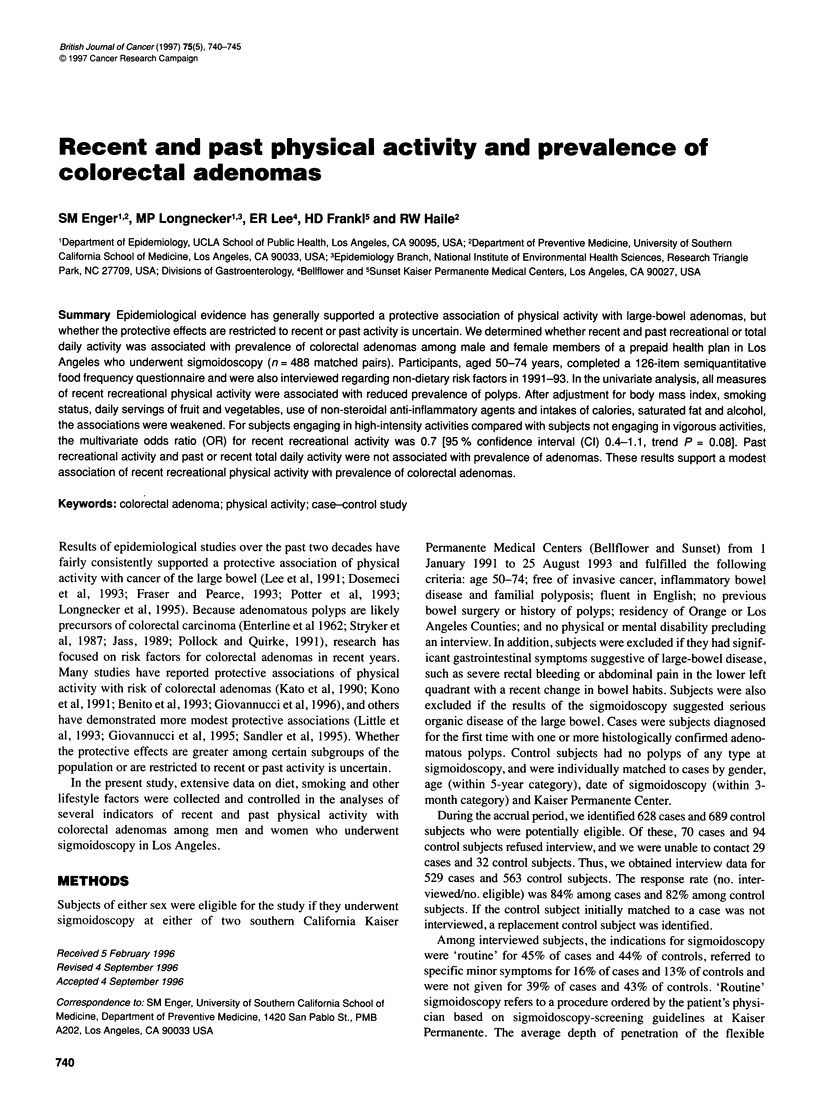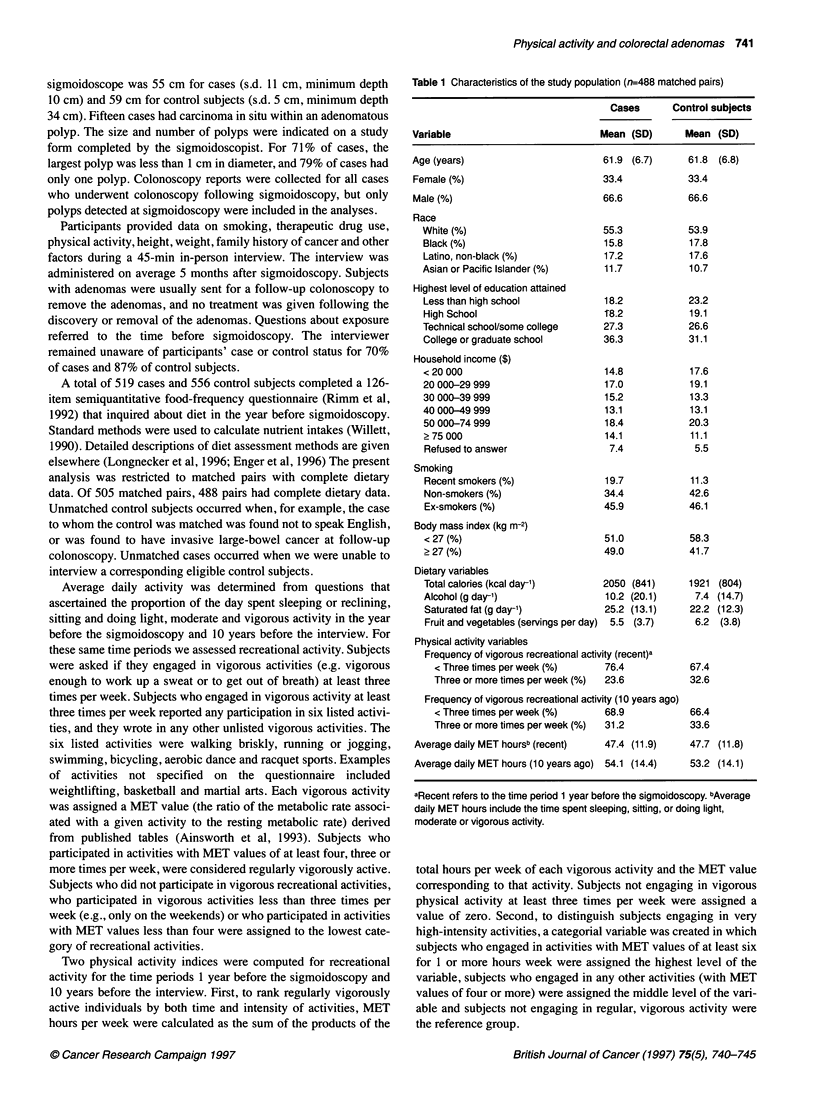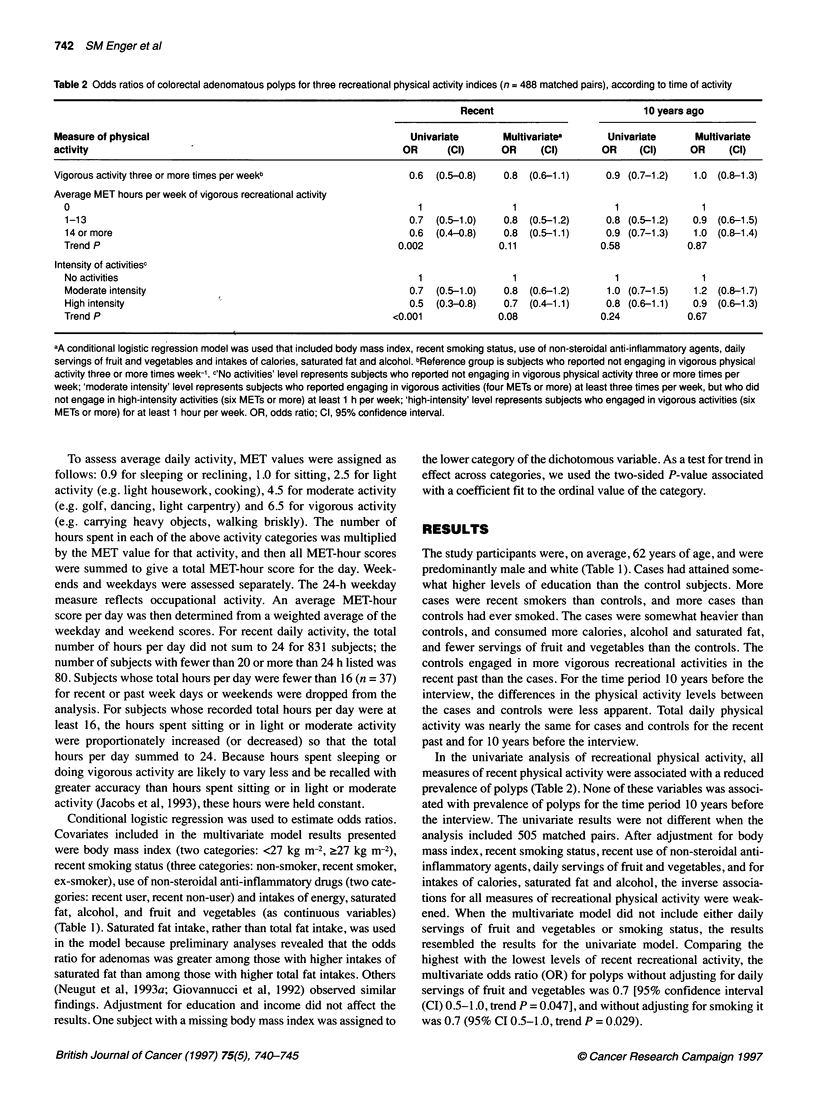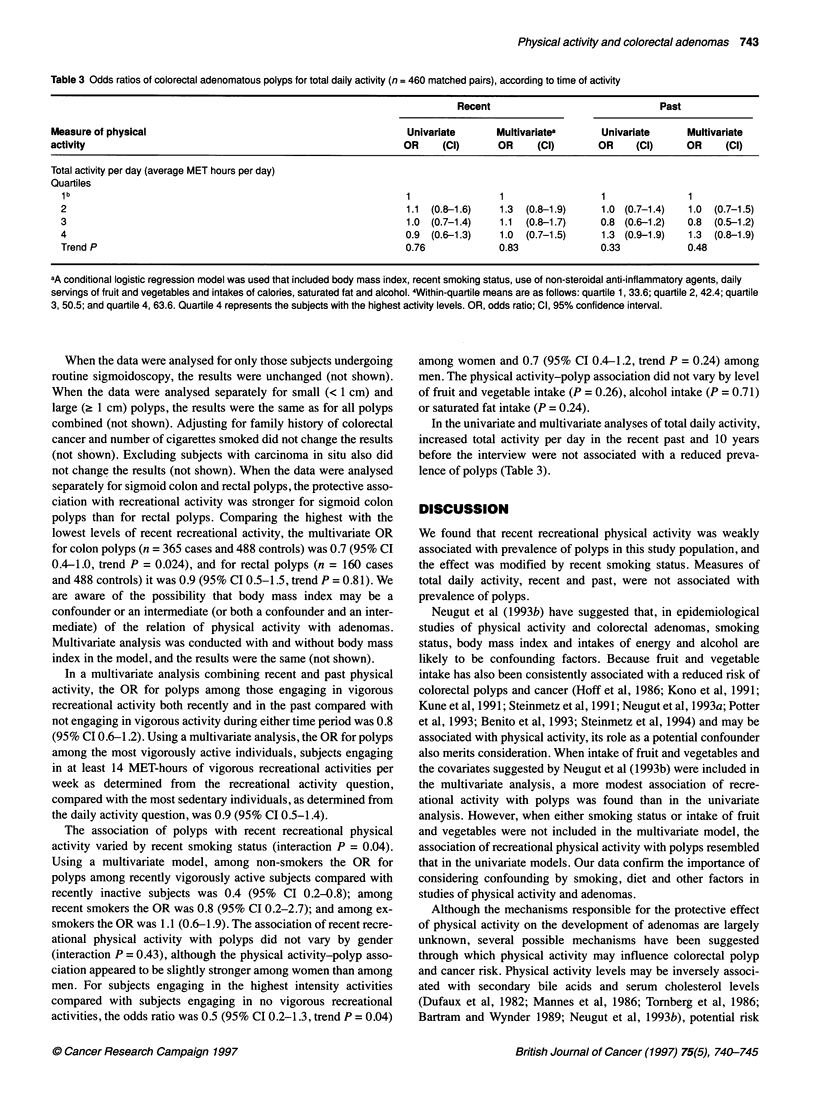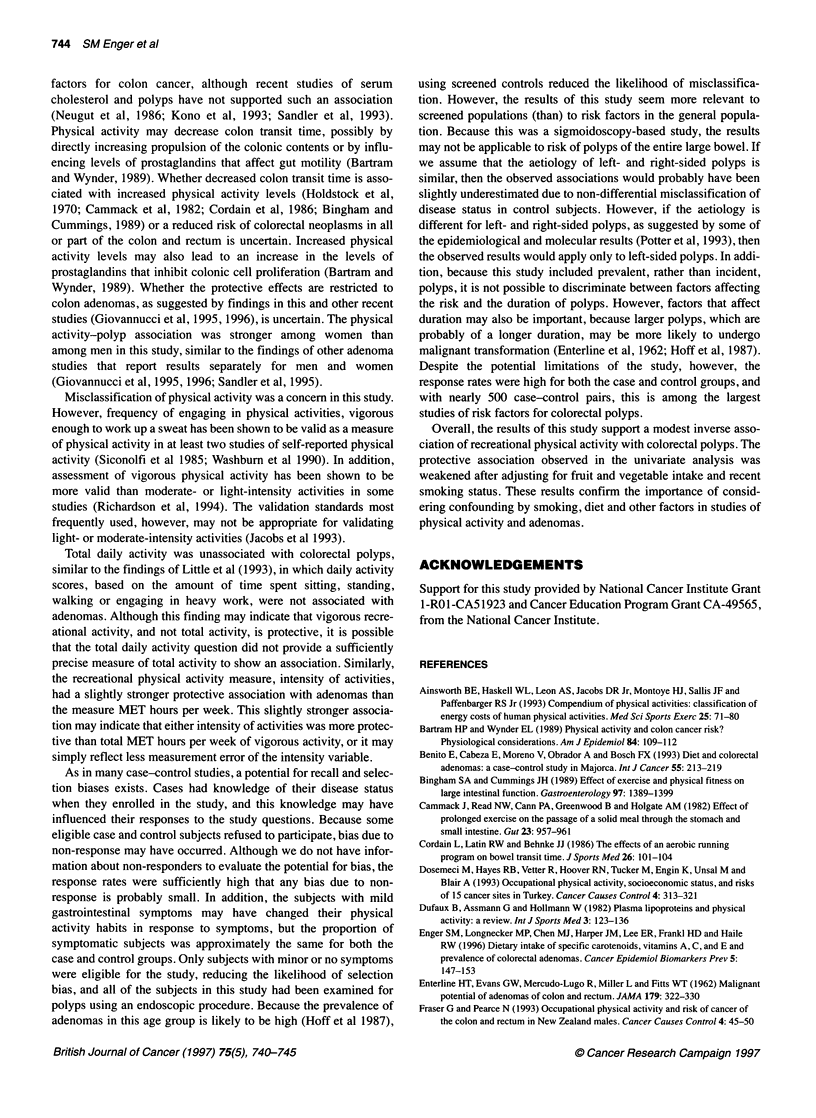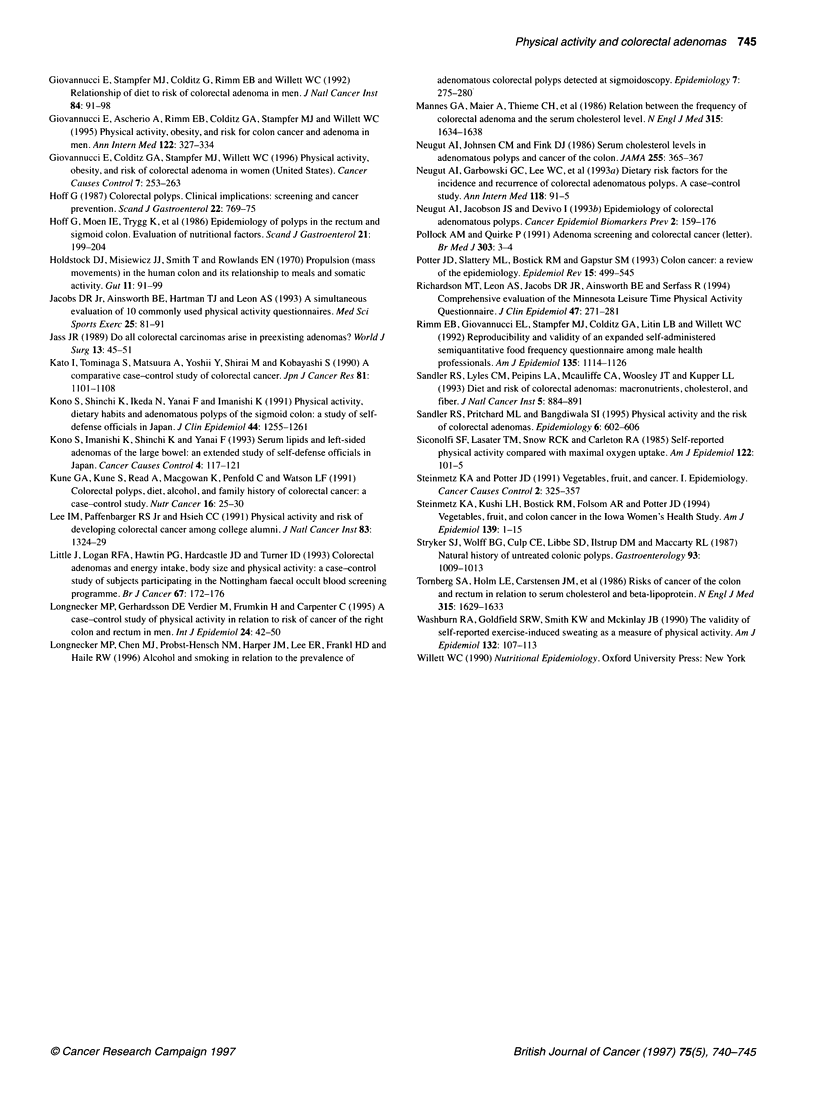# Recent and past physical activity and prevalence of colorectal adenomas.

**DOI:** 10.1038/bjc.1997.131

**Published:** 1997

**Authors:** S. M. Enger, M. P. Longnecker, E. R. Lee, H. D. Frankl, R. W. Haile

**Affiliations:** Department of Epidemiology, UCLA School of Public Health, Los Angeles, CA 90095, USA.

## Abstract

Epidemiological evidence has generally supported a protective association of physical activity with large-bowel adenomas, but whether the protective effects are restricted to recent or past activity is uncertain. We determined whether recent and past recreational or total daily activity was associated with prevalence of colorectal adenomas among male and female members of a prepaid health plan in Los Angeles who underwent sigmoidoscopy (n = 488 matched pairs). Participants, aged 50-74 years, completed a 126-item semiquantitative food frequency questionnaire and were also interviewed regarding non-dietary risk factors in 1991-93. In the univariate analysis, all measures of recent recreational physical activity were associated with reduced prevalence of polyps. After adjustment for body mass index, smoking status, daily servings of fruit and vegetables, use of non-steroidal anti-inflammatory agents and intakes of calories, saturated fat and alcohol, the associations were weakened. For subjects engaging in high-intensity activities compared with subjects not engaging in vigorous activities, the multivariate odds ratio (OR) for recent recreational activity was 0.7 [95% confidence interval (CI) 0.4-1.1, trend P = 0.08]. Past recreational activity and past or recent total daily activity were not associated with prevalence of adenomas. These results support a modest association of recent recreational physical activity with prevalence of colorectal adenomas.


British Journal of Cancer (1997) 75(5), 740–745
© 1997 Cancer Research Campaign

### Recent and past physical activity and prevalence of colorectal adenomas

**SM Enger[1,2], MP Longnecker[1,3], ER Lee[4], HD Frankl[5] and RW Haile[2]**

[1]Department of Epidemiology, UCLA School of Public Health, Los Angeles, CA 90095, USA; [2]Department of Preventive Medicine, University of Southern California School of Medicine, Los Angeles, CA 90033, USA; [3]Epidemiology Branch, National Institute of Environmental Health Sciences, Research Triangle Park, NC 27709, USA; Divisions of Gastroenterology, [4]Bellflower and [5]Sunset Kaiser Permanente Medical Centers, Los Angeles, CA 90027, USA

**Summary** Epidemiological evidence has generally supported a protective association of physical activity with large-bowel adenomas, but whether the protective effects are restricted to recent or past activity is uncertain. We determined whether recent and past recreational or total daily activity was associated with prevalence of colorectal adenomas among male and female members of a prepaid health plan in Los Angeles who underwent sigmoidoscopy ($n = 488$ matched pairs). Participants, aged 50–74 years, completed a 126-item semiquantitative food frequency questionnaire and were also interviewed regarding non-dietary risk factors in 1991–93. In the univariate analysis, all measures of recent recreational physical activity were associated with reduced prevalence of polyps. After adjustment for body mass index, smoking status, daily servings of fruit and vegetables, use of non-steroidal anti-inflammatory agents and intakes of calories, saturated fat and alcohol, the associations were weakened. For subjects engaging in high-intensity activities compared with subjects not engaging in vigorous activities, the multivariate odds ratio (OR) for recent recreational activity was 0.7 [95 % confidence interval (CI) 0.4–1.1, trend $P = 0.08$]. Past recreational activity and past or recent total daily activity were not associated with prevalence of adenomas. These results support a modest association of recent recreational physical activity with prevalence of colorectal adenomas.

**Keywords:** colorectal adenoma; physical activity; case–control study

Results of epidemiological studies over the past two decades have fairly consistently supported a protective association of physical activity with cancer of the large bowel (Lee et al, 1991; Dosemeci et al, 1993; Fraser and Pearce, 1993; Potter et al, 1993; Longnecker et al, 1995). Because adenomatous polyps are likely precursors of colorectal carcinoma (Enterline et al 1962; Stryker et al, 1987; Jass, 1989; Pollock and Quirke, 1991), research has focused on risk factors for colorectal adenomas in recent years. Many studies have reported protective associations of physical activity with risk of colorectal adenomas (Kato et al, 1990; Kono et al, 1991; Benito et al, 1993; Giovannucci et al, 1996), and others have demonstrated more modest protective associations (Little et al, 1993; Giovannucci et al, 1995; Sandler et al, 1995). Whether the protective effects are greater among certain subgroups of the population or are restricted to recent or past activity is uncertain.

In the present study, extensive data on diet, smoking and other lifestyle factors were collected and controlled in the analyses of several indicators of recent and past physical activity with colorectal adenomas among men and women who underwent sigmoidoscopy in Los Angeles.

### METHODS

Subjects of either sex were eligible for the study if they underwent sigmoidoscopy at either of two southern California Kaiser Permanente Medical Centers (Bellflower and Sunset) from 1 January 1991 to 25 August 1993 and fulfilled the following criteria: age 50–74; free of invasive cancer, inflammatory bowel disease and familial polyposis; fluent in English; no previous bowel surgery or history of polyps; residency of Orange or Los Angeles Counties; and no physical or mental disability precluding an interview. In addition, subjects were excluded if they had significant gastrointestinal symptoms suggestive of large-bowel disease, such as severe rectal bleeding or abdominal pain in the lower left quadrant with a recent change in bowel habits. Subjects were also excluded if the results of the sigmoidoscopy suggested serious organic disease of the large bowel. Cases were subjects diagnosed for the first time with one or more histologically confirmed adenomatous polyps. Control subjects had no polyps of any type at sigmoidoscopy, and were individually matched to cases by gender, age (within 5-year category), date of sigmoidoscopy (within 3-month category) and Kaiser Permanente Center.

During the accrual period, we identified 628 cases and 689 control subjects who were potentially eligible. Of these, 70 cases and 94 control subjects refused interview, and we were unable to contact 29 cases and 32 control subjects. Thus, we obtained interview data for 529 cases and 563 control subjects. The response rate (no. interviewed/no. eligible) was 84% among cases and 82% among control subjects. If the control subject initially matched to a case was not interviewed, a replacement control subject was identified.

Among interviewed subjects, the indications for sigmoidoscopy were 'routine' for 45% of cases and 44% of controls, referred to specific minor symptoms for 16% of cases and 13% of controls and were not given for 39% of cases and 43% of controls. 'Routine' sigmoidoscopy refers to a procedure ordered by the patient's physician based on sigmoidoscopy-screening guidelines at Kaiser Permanente. The average depth of penetration of the flexible

*Received 5 February 1996*
*Revised 4 September 1996*
*Accepted 4 September 1996*

*Correspondence to:* SM Enger, University of Southern California School of Medicine, Department of Preventive Medicine, 1420 San Pablo St., PMB A202, Los Angeles, CA 90033 USA

sigmoidoscope was 55 cm for cases (s.d. 11 cm, minimum depth 10 cm) and 59 cm for control subjects (s.d. 5 cm, minimum depth 34 cm). Fifteen cases had carcinoma in situ within an adenomatous polyp. The size and number of polyps were indicated on a study form completed by the sigmoidoscopist. For 71% of cases, the largest polyp was less than 1 cm in diameter, and 79% of cases had only one polyp. Colonoscopy reports were collected for all cases who underwent colonoscopy following sigmoidoscopy, but only polyps detected at sigmoidoscopy were included in the analyses.

Participants provided data on smoking, therapeutic drug use, physical activity, height, weight, family history of cancer and other factors during a 45-min in-person interview. The interview was administered on average 5 months after sigmoidoscopy. Subjects with adenomas were usually sent for a follow-up colonoscopy to remove the adenomas, and no treatment was given following the discovery or removal of the adenomas. Questions about exposure referred to the time before sigmoidoscopy. The interviewer remained unaware of participants' case or control status for 70% of cases and 87% of control subjects.

A total of 519 cases and 556 control subjects completed a 126-item semiquantitative food-frequency questionnaire (Rimm et al, 1992) that inquired about diet in the year before sigmoidoscopy. Standard methods were used to calculate nutrient intakes (Willett, 1990). Detailed descriptions of diet assessment methods are given elsewhere (Longnecker et al, 1996; Enger et al, 1996) The present analysis was restricted to matched pairs with complete dietary data. Of 505 matched pairs, 488 pairs had complete dietary data. Unmatched control subjects occurred when, for example, the case to whom the control was matched was found not to speak English, or was found to have invasive large-bowel cancer at follow-up colonoscopy. Unmatched cases occurred when we were unable to interview a corresponding eligible control subjects.

Average daily activity was determined from questions that ascertained the proportion of the day spent sleeping or reclining, sitting and doing light, moderate and vigorous activity in the year before the sigmoidoscopy and 10 years before the interview. For these same time periods we assessed recreational activity. Subjects were asked if they engaged in vigorous activities (e.g. vigorous enough to work up a sweat or to get out of breath) at least three times per week. Subjects who engaged in vigorous activity at least three times per week reported any participation in six listed activities, and they wrote in any other unlisted vigorous activities. The six listed activities were walking briskly, running or jogging, swimming, bicycling, aerobic dance and racquet sports. Examples of activities not specified on the questionnaire included weightlifting, basketball and martial arts. Each vigorous activity was assigned a MET value (the ratio of the metabolic rate associated with a given activity to the resting metabolic rate) derived from published tables (Ainsworth et al, 1993). Subjects who participated in activities with MET values of at least four, three or more times per week, were considered regularly vigorously active. Subjects who did not participate in vigorous recreational activities, who participated in vigorous activities less than three times per week (e.g., only on the weekends) or who participated in activities with MET values less than four were assigned to the lowest category of recreational activities.

Two physical activity indices were computed for recreational activity for the time periods 1 year before the sigmoidoscopy and 10 years before the interview. First, to rank regularly vigorously active individuals by both time and intensity of activities, MET hours per week were calculated as the sum of the products of the total hours per week of each vigorous activity and the MET value corresponding to that activity. Subjects not engaging in vigorous physical activity at least three times per week were assigned a value of zero. Second, to distinguish subjects engaging in very high-intensity activities, a categorial variable was created in which subjects who engaged in activities with MET values of at least six for 1 or more hours week were assigned the highest level of the variable, subjects who engaged in any other activities (with MET values of four or more) were assigned the middle level of the variable and subjects not engaging in regular, vigorous activity were the reference group.

**Table 1** Characteristics of the study population (*n*=488 matched pairs)

| | **Cases** | | **Control subjects** | |
|---|---|---|---|---|
| **Variable** | **Mean** | **(SD)** | **Mean** | **(SD)** |
| Age (years) | 61.9 | (6.7) | 61.8 | (6.8) |
| Female (%) | 33.4 | | 33.4 | |
| Male (%) | 66.6 | | 66.6 | |
| Race | | | | |
| White (%) | 55.3 | | 53.9 | |
| Black (%) | 15.8 | | 17.8 | |
| Latino, non-black (%) | 17.2 | | 17.6 | |
| Asian or Pacific Islander (%) | 11.7 | | 10.7 | |
| Highest level of education attained | | | | |
| Less than high school | 18.2 | | 23.2 | |
| High School | T8.2 | | 19.1 | |
| Technical school/some college | 27.3 | | 26.6 | |
| College or graduate school | 36.3 | | 31.1 | |
| Household income ($) | | | | |
| < 20 000 | 14.8 | | 17.6 | |
| 20 000–29 999 | 17.0 | | 19.1 | |
| 30 000–39 999 | 15.2 | | 13.3 | |
| 40 000–49 999 | 13.1 | | 13.1 | |
| 50 000–74 999 | 18.4 | | 20.3 | |
| ≥ 75 000 | 14.1 | | 11.1 | |
| Refused to answer | 7.4 | | 5.5 | |
| Smoking | | | | |
| Recent smokers (%) | 19.7 | | 11.3 | |
| Non-smokers (%) | 34.4 | | 42.6 | |
| Ex-smokers (%) | 45.9 | | 46.1 | |
| Body mass index (kg $m^{-2}$) | | | | |
| < 27 (%) | 51.0 | | 58.3 | |
| ≥ 27 (%) | 49.0 | | 41.7 | |
| Dietary variables | | | | |
| Total calories (kcal $day^{-1}$) | 2050 | (841) | 1921 | (804) |
| Alcohol (g $day^{-1}$) | 10.2 | (20.1) | 7.4 | (14.7) |
| Saturated fat (g $day^{-1}$) | 25.2 | (13.1) | 22.2 | (12.3) |
| Fruit and vegetables (servings per day) | 5.5 | (3.7) | 6.2 | (3.8) |
| Physical activity variables | | | | |
| Frequency of vigorous recreational activity (recent)[a] | | | | |
| < Three times per week (%) | 76.4 | | 67.4 | |
| Three or more times per week (%) | 23.6 | | 32.6 | |
| Frequency of vigorous recreational activity (10 years ago) | | | | |
| < Three times per week (%) | 68.9 | | 66.4 | |
| Three or more times per week (%) | 31.2 | | 33.6 | |
| Average daily MET hours[b] (recent) | 47.4 | (11.9) | 47.7 | (11.8) |
| Average daily MET hours (10 years ago) | 54.1 | (14.4) | 53.2 | (14.1) |

[a]Recent refers to the time period 1 year before the sigmoidoscopy. [b]Average daily MET hours include the time spent sleeping, sitting, or doing light, moderate or vigorous activity.

*© Cancer Research Campaign 1997*

Table 2 Odds ratios of colorectal adenomatous polyps for three recreational physical activity indices ($n$ = 488 matched pairs), according to time of activity

| Measure of physical activity | Recent | | | | 10 years ago | | | |
|---|---|---|---|---|---|---|---|---|
| | Univariate | | Multivariate[a] | | Univariate | | Multivariate | |
| | OR | (CI) | OR | (CI) | OR | (CI) | OR | (CI) |
| Vigorous activity three or more times per week[b] | 0.6 | (0.5–0.8) | 0.8 | (0.6–1.1) | 0.9 | (0.7–1.2) | 1.0 | (0.8–1.3) |
| Average MET hours per week of vigorous recreational activity | | | | | | | | |
| 0 | 1 | | 1 | | 1 | | 1 | |
| 1–13 | 0.7 | (0.5–1.0) | 0.8 | (0.5–1.2) | 0.8 | (0.5–1.2) | 0.9 | (0.6–1.5) |
| 14 or more | 0.6 | (0.4–0.8) | 0.8 | (0.5–1.1) | 0.9 | (0.7–1.3) | 1.0 | (0.8–1.4) |
| Trend *P* | 0.002 | | 0.11 | | 0.58 | | 0.87 | |
| Intensity of activities[c] | | | | | | | | |
| No activities | 1 | | 1 | | 1 | | 1 | |
| Moderate intensity | 0.7 | (0.5–1.0) | 0.8 | (0.6–1.2) | 1.0 | (0.7–1.5) | 1.2 | (0.8–1.7) |
| High intensity | 0.5 | (0.3–0.8) | 0.7 | (0.4–1.1) | 0.8 | (0.6–1.1) | 0.9 | (0.6–1.3) |
| Trend *P* | <0.001 | | 0.08 | | 0.24 | | 0.67 | |

[a]A conditional logistic regression model was used that included body mass index, recent smoking status, use of non-steroidal anti-inflammatory agents, daily servings of fruit and vegetables and intakes of calories, saturated fat and alcohol. [b]Reference group is subjects who reported not engaging in vigorous physical activity three or more times week$^{-1}$. [c]'No activities' level represents subjects who reported not engaging in vigorous physical activity three or more times per week; 'moderate intensity' level represents subjects who reported engaging in vigorous activities (four METs or more) at least three times per week, but who did not engage in high-intensity activities (six METs or more) at least 1 h per week; 'high-intensity' level represents subjects who engaged in vigorous activities (six METs or more) for at least 1 hour per week. OR, odds ratio; CI, 95% confidence interval.

To assess average daily activity, MET values were assigned as follows: 0.9 for sleeping or reclining, 1.0 for sitting, 2.5 for light activity (e.g. light housework, cooking), 4.5 for moderate activity (e.g. golf, dancing, light carpentry) and 6.5 for vigorous activity (e.g. carrying heavy objects, walking briskly). The number of hours spent in each of the above activity categories was multiplied by the MET value for that activity, and then all MET-hour scores were summed to give a total MET-hour score for the day. Weekends and weekdays were assessed separately. The 24-h weekday measure reflects occupational activity. An average MET-hour score per day was then determined from a weighted average of the weekday and weekend scores. For recent daily activity, the total number of hours per day did not sum to 24 for 831 subjects; the number of subjects with fewer than 20 or more than 24 h listed was 80. Subjects whose total hours per day were fewer than 16 ($n$ = 37) for recent or past week days or weekends were dropped from the analysis. For subjects whose recorded total hours per day were at least 16, the hours spent sitting or in light or moderate activity were proportionately increased (or decreased) so that the total hours per day summed to 24. Because hours spent sleeping or doing vigorous activity are likely to vary less and be recalled with greater accuracy than hours spent sitting or in light or moderate activity (Jacobs et al, 1993), these hours were held constant.

Conditional logistic regression was used to estimate odds ratios. Covariates included in the multivariate model results presented were body mass index (two categories: <27 kg m$^{-2}$, ≥27 kg m$^{-2}$), recent smoking status (three categories: non-smoker, recent smoker, ex-smoker), use of non-steroidal anti-inflammatory drugs (two categories: recent user, recent non-user) and intakes of energy, saturated fat, alcohol, and fruit and vegetables (as continuous variables) (Table 1). Saturated fat intake, rather than total fat intake, was used in the model because preliminary analyses revealed that the odds ratio for adenomas was greater among those with higher intakes of saturated fat than among those with higher total fat intakes. Others (Neugut et al, 1993*a*; Giovannucci et al, 1992) observed similar findings. Adjustment for education and income did not affect the results. One subject with a missing body mass index was assigned to the lower category of the dichotomous variable. As a test for trend in effect across categories, we used the two-sided *P*-value associated with a coefficient fit to the ordinal value of the category.

### RESULTS

The study participants were, on average, 62 years of age, and were predominantly male and white (Table 1). Cases had attained somewhat higher levels of education than the control subjects. More cases were recent smokers than controls, and more cases than controls had ever smoked. The cases were somewhat heavier than controls, and consumed more calories, alcohol and saturated fat, and fewer servings of fruit and vegetables than the controls. The controls engaged in more vigorous recreational activities in the recent past than the cases. For the time period 10 years before the interview, the differences in the physical activity levels between the cases and controls were less apparent. Total daily physical activity was nearly the same for cases and controls for the recent past and for 10 years before the interview.

In the univariate analysis of recreational physical activity, all measures of recent physical activity were associated with a reduced prevalence of polyps (Table 2). None of these variables was associated with prevalence of polyps for the time period 10 years before the interview. The univariate results were not different when the analysis included 505 matched pairs. After adjustment for body mass index, recent smoking status, recent use of non-steroidal anti-inflammatory agents, daily servings of fruit and vegetables, and for intakes of calories, saturated fat and alcohol, the inverse associations for all measures of recreational physical activity were weakened. When the multivariate model did not include either daily servings of fruit and vegetables or smoking status, the results resembled the results for the univariate model. Comparing the highest with the lowest levels of recent recreational activity, the multivariate odds ratio (OR) for polyps without adjusting for daily servings of fruit and vegetables was 0.7 [95% confidence interval (CI) 0.5–1.0, trend $P$ = 0.047], and without adjusting for smoking it was 0.7 (95% CI 0.5–1.0, trend $P$ = 0.029).

© Cancer Research Campaign 1997

**Table 3** Odds ratios of colorectal adenomatous polyps for total daily activity ($n$ = 460 matched pairs), according to time of activity

| Measure of physical activity | Recent | | | | Past | | | |
|---|---|---|---|---|---|---|---|---|
| | Univariate | | Multivariate[a] | | Univariate | | Multivariate | |
| | OR | (CI) | OR | (CI) | OR | (CI) | OR | (CI) |
| Total activity per day (average MET hours per day) | | | | | | | | |
| Quartiles | | | | | | | | |
| 1[b] | 1 | | 1 | | 1 | | 1 | |
| 2 | 1.1 | (0.8–1.6) | 1.3 | (0.8–1.9) | 1.0 | (0.7–1.4) | 1.0 | (0.7–1.5) |
| 3 | 1.0 | (0.7–1.4) | 1.1 | (0.8–1.7) | 0.8 | (0.6–1.2) | 0.8 | (0.5–1.2) |
| 4 | 0.9 | (0.6–1.3) | 1.0 | (0.7–1.5) | 1.3 | (0.9–1.9) | 1.3 | (0.8–1.9) |
| Trend $P$ | 0.76 | | 0.83 | | 0.33 | | 0.48 | |

[a]A conditional logistic regression model was used that included body mass index, recent smoking status, use of non-steroidal anti-inflammatory agents, daily servings of fruit and vegetables and intakes of calories, saturated fat and alcohol. [d]Within-quartile means are as follows: quartile 1, 33.6; quartile 2, 42.4; quartile 3, 50.5; and quartile 4, 63.6. Quartile 4 represents the subjects with the highest activity levels. OR, odds ratio; CI, 95% confidence interval.

When the data were analysed for only those subjects undergoing routine sigmoidoscopy, the results were unchanged (not shown). When the data were analysed separately for small (< 1 cm) and large (≥ 1 cm) polyps, the results were the same as for all polyps combined (not shown). Adjusting for family history of colorectal cancer and number of cigarettes smoked did not change the results (not shown). Excluding subjects with carcinoma in situ also did not change the results (not shown). When the data were analysed separately for sigmoid colon and rectal polyps, the protective association with recreational activity was stronger for sigmoid colon polyps than for rectal polyps. Comparing the highest with the lowest levels of recent recreational activity, the multivariate OR for colon polyps ($n$ = 365 cases and 488 controls) was 0.7 (95% CI 0.4–1.0, trend $P$ = 0.024), and for rectal polyps ($n$ = 160 cases and 488 controls) it was 0.9 (95% CI 0.5–1.5, trend $P$ = 0.81). We are aware of the possibility that body mass index may be a confounder or an intermediate (or both a confounder and an intermediate) of the relation of physical activity with adenomas. Multivariate analysis was conducted with and without body mass index in the model, and the results were the same (not shown).

In a multivariate analysis combining recent and past physical activity, the OR for polyps among those engaging in vigorous recreational activity both recently and in the past compared with not engaging in vigorous activity during either time period was 0.8 (95% CI 0.6–1.2). Using a multivariate analysis, the OR for polyps among the most vigorously active individuals, subjects engaging in at least 14 MET-hours of vigorous recreational activities per week as determined from the recreational activity question, compared with the most sedentary individuals, as determined from the daily activity question, was 0.9 (95% CI 0.5–1.4).

The association of polyps with recent recreational physical activity varied by recent smoking status (interaction $P$ = 0.04). Using a multivariate model, among non-smokers the OR for polyps among recently vigorously active subjects compared with recently inactive subjects was 0.4 (95% CI 0.2–0.8); among recent smokers the OR was 0.8 (95% CI 0.2–2.7); and among ex-smokers the OR was 1.1 (0.6–1.9). The association of recent recreational physical activity with polyps did not vary by gender (interaction $P$ = 0.43), although the physical activity–polyp association appeared to be slightly stronger among women than among men. For subjects engaging in the highest intensity activities compared with subjects engaging in no vigorous recreational activities, the odds ratio was 0.5 (95% CI 0.2–1.3, trend $P$ = 0.04) among women and 0.7 (95% CI 0.4–1.2, trend $P$ = 0.24) among men. The physical activity–polyp association did not vary by level of fruit and vegetable intake ($P$ = 0.26), alcohol intake ($P$ = 0.71) or saturated fat intake ($P$ = 0.24).

In the univariate and multivariate analyses of total daily activity, increased total activity per day in the recent past and 10 years before the interview were not associated with a reduced prevalence of polyps (Table 3).

### DISCUSSION

We found that recent recreational physical activity was weakly associated with prevalence of polyps in this study population, and the effect was modified by recent smoking status. Measures of total daily activity, recent and past, were not associated with prevalence of polyps.

Neugut et al (1993*b*) have suggested that, in epidemiological studies of physical activity and colorectal adenomas, smoking status, body mass index and intakes of energy and alcohol are likely to be confounding factors. Because fruit and vegetable intake has also been consistently associated with a reduced risk of colorectal polyps and cancer (Hoff et al, 1986; Kono et al, 1991; Kune et al, 1991; Steinmetz et al, 1991; Neugut et al, 1993*a*; Potter et al, 1993; Benito et al, 1993; Steinmetz et al, 1994) and may be associated with physical activity, its role as a potential confounder also merits consideration. When intake of fruit and vegetables and the covariates suggested by Neugut et al (1993b) were included in the multivariate analysis, a more modest association of recreational activity with polyps was found than in the univariate analysis. However, when either smoking status or intake of fruit and vegetables were not included in the multivariate model, the association of recreational physical activity with polyps resembled that in the univariate models. Our data confirm the importance of considering confounding by smoking, diet and other factors in studies of physical activity and adenomas.

Although the mechanisms responsible for the protective effect of physical activity on the development of adenomas are largely unknown, several possible mechanisms have been suggested through which physical activity may influence colorectal polyp and cancer risk. Physical activity levels may be inversely associated with secondary bile acids and serum cholesterol levels (Dufaux et al, 1982; Mannes et al, 1986; Tornberg et al, 1986; Bartram and Wynder 1989; Neugut et al, 1993*b*), potential risk

© Cancer Research Campaign 1997

factors for colon cancer, although recent studies of serum cholesterol and polyps have not supported such an association (Neugut et al, 1986; Kono et al, 1993; Sandler et al, 1993). Physical activity may decrease colon transit time, possibly by directly increasing propulsion of the colonic contents or by influencing levels of prostaglandins that affect gut motility (Bartram and Wynder, 1989). Whether decreased colon transit time is associated with increased physical activity levels (Holdstock et al, 1970; Cammack et al, 1982; Cordain et al, 1986; Bingham and Cummings, 1989) or a reduced risk of colorectal neoplasms in all or part of the colon and rectum is uncertain. Increased physical activity levels may also lead to an increase in the levels of prostaglandins that inhibit colonic cell proliferation (Bartram and Wynder, 1989). Whether the protective effects are restricted to colon adenomas, as suggested by findings in this and other recent studies (Giovannucci et al, 1995, 1996), is uncertain. The physical activity–polyp association was stronger among women than among men in this study, similar to the findings of other adenoma studies that report results separately for men and women (Giovannucci et al, 1995, 1996; Sandler et al, 1995).

Misclassification of physical activity was a concern in this study. However, frequency of engaging in physical activities, vigorous enough to work up a sweat has been shown to be valid as a measure of physical activity in at least two studies of self-reported physical activity (Siconolfi et al 1985; Washburn et al 1990). In addition, assessment of vigorous physical activity has been shown to be more valid than moderate- or light-intensity activities in some studies (Richardson et al, 1994). The validation standards most frequently used, however, may not be appropriate for validating light- or moderate-intensity activities (Jacobs et al 1993).

Total daily activity was unassociated with colorectal polyps, similar to the findings of Little et al (1993), in which daily activity scores, based on the amount of time spent sitting, standing, walking or engaging in heavy work, were not associated with adenomas. Although this finding may indicate that vigorous recreational activity, and not total activity, is protective, it is possible that the total daily activity question did not provide a sufficiently precise measure of total activity to show an association. Similarly, the recreational physical activity measure, intensity of activities, had a slightly stronger protective association with adenomas than the measure MET hours per week. This slightly stronger association may indicate that either intensity of activities was more protective than total MET hours per week of vigorous activity, or it may simply reflect less measurement error of the intensity variable.

As in many case–control studies, a potential for recall and selection biases exists. Cases had knowledge of their disease status when they enrolled in the study, and this knowledge may have influenced their responses to the study questions. Because some eligible case and control subjects refused to participate, bias due to non-response may have occurred. Although we do not have information about non-responders to evaluate the potential for bias, the response rates were sufficiently high that any bias due to non-response is probably small. In addition, the subjects with mild gastrointestinal symptoms may have changed their physical activity habits in response to symptoms, but the proportion of symptomatic subjects was approximately the same for both the case and control groups. Only subjects with minor or no symptoms were eligible for the study, reducing the likelihood of selection bias, and all of the subjects in this study had been examined for polyps using an endoscopic procedure. Because the prevalence of adenomas in this age group is likely to be high (Hoff et al 1987), using screened controls reduced the likelihood of misclassification. However, the results of this study seem more relevant to screened populations (than) to risk factors in the general population. Because this was a sigmoidoscopy-based study, the results may not be applicable to risk of polyps of the entire large bowel. If we assume that the aetiology of left- and right-sided polyps is similar, then the observed associations would probably have been slightly underestimated due to non-differential misclassification of disease status in control subjects. However, if the aetiology is different for left- and right-sided polyps, as suggested by some of the epidemiological and molecular results (Potter et al, 1993), then the observed results would apply only to left-sided polyps. In addition, because this study included prevalent, rather than incident, polyps, it is not possible to discriminate between factors affecting the risk and the duration of polyps. However, factors that affect duration may also be important, because larger polyps, which are probably of a longer duration, may be more likely to undergo malignant transformation (Enterline et al, 1962; Hoff et al, 1987). Despite the potential limitations of the study, however, the response rates were high for both the case and control groups, and with nearly 500 case–control pairs, this is among the largest studies of risk factors for colorectal polyps.

Overall, the results of this study support a modest inverse association of recreational physical activity with colorectal polyps. The protective association observed in the univariate analysis was weakened after adjusting for fruit and vegetable intake and recent smoking status. These results confirm the importance of considering confounding by smoking, diet and other factors in studies of physical activity and adenomas.

### ACKNOWLEDGEMENTS

Support for this study provided by National Cancer Institute Grant 1-R01-CA51923 and Cancer Education Program Grant CA-49565, from the National Cancer Institute.

### REFERENCES

Ainsworth BE, Haskell WL, Leon AS, Jacobs DR Jr, Montoye HJ, Sallis JF and Paffenbarger RS Jr (1993) Compendium of physical activities: classification of energy costs of human physical activities. *Med Sci Sports Exerc* **25**: 71–80

Bartram HP and Wynder EL (1989) Physical activity and colon cancer risk? Physiological considerations. *Am J Epidemiol* **84**: 109–112

Benito E, Cabeza E, Moreno V, Obrador A and Bosch FX (1993) Diet and colorectal adenomas: a case–control study in Majorca. *Int J Cancer* **55**: 213–219

Bingham SA and Cummings JH (1989) Effect of exercise and physical fitness on large intestinal function. *Gastroenterology* **97**: 1389–1399

Cammack J, Read NW, Cann PA, Greenwood B and Holgate AM (1982) Effect of prolonged exercise on the passage of a solid meal through the stomach and small intestine. *Gut* **23**: 957–961

Cordain L, Latin RW and Behnke JJ (1986) The effects of an aerobic running program on bowel transit time. *J Sports Med* **26**: 101–104

Dosemeci M, Hayes RB, Vetter R, Hoover RN, Tucker M, Engin K, Unsal M and Blair A (1993) Occupational physical activity, socioeconomic status, and risks of 15 cancer sites in Turkey. *Cancer Causes Control* **4**: 313–321

Dufaux B, Assmann G and Hollmann W (1982) Plasma lipoproteins and physical activity: a review. *Int J Sports Med* **3**: 123–136

Enger SM, Longnecker MP, Chen MJ, Harper JM, Lee ER, Frankl HD and Haile RW (1996) Dietary intake of specific carotenoids, vitamins A, C, and E and prevalence of colorectal adenomas. *Cancer Epidemiol Biomarkers Prev* **5**: 147–153

Enterline HT, Evans GW, Mercudo-Lugo R, Miller L and Fitts WT (1962) Malignant potential of adenomas of colon and rectum. *JAMA* **179**: 322–330

Fraser G and Pearce N (1993) Occupational physical activity and risk of cancer of the colon and rectum in New Zealand males. *Cancer Causes Control* **4**: 45–50

© Cancer Research Campaign 1997

Giovannucci E, Stampfer MJ, Colditz G, Rimm EB and Willett WC (1992) Relationship of diet to risk of colorectal adenoma in men. *J Natl Cancer Inst* **84**: 91–98

Giovannucci E, Ascherio A, Rimm EB, Colditz GA, Stampfer MJ and Willett WC (1995) Physical activity, obesity, and risk for colon cancer and adenoma in men. *Ann Intern Med* **122**: 327–334

Giovannucci E, Colditz GA, Stampfer MJ, Willett WC (1996) Physical activity, obesity, and risk of colorectal adenoma in women (United States). *Cancer Causes Control* **7**: 253–263

Hoff G (1987) Colorectal polyps. Clinical implications: screening and cancer prevention. *Scand J Gastroenterol* **22**: 769–75

Hoff G, Moen IE, Trygg K, et al (1986) Epidemiology of polyps in the rectum and sigmoid colon. Evaluation of nutritional factors. *Scand J Gastroenterol* **21**: 199–204

Holdstock DJ, Misiewicz JJ, Smith T and Rowlands EN (1970) Propulsion (mass movements) in the human colon and its relationship to meals and somatic activity. *Gut* **11**: 91–99

Jacobs DR Jr, Ainsworth BE, Hartman TJ and Leon AS (1993) A simultaneous evaluation of 10 commonly used physical activity questionnaires. *Med Sci Sports Exerc* **25**: 81–91

Jass JR (1989) Do all colorectal carcinomas arise in preexisting adenomas? *World J Surg* **13**: 45–51

Kato I, Tominaga S, Matsuura A, Yoshii Y, Shirai M and Kobayashi S (1990) A comparative case–control study of colorectal cancer. *Jpn J Cancer Res* **81**: 1101–1108

Kono S, Shinchi K, Ikeda N, Yanai F and Imanishi K (1991) Physical activity, dietary habits and adenomatous polyps of the sigmoid colon: a study of self-defense officials in Japan. *J Clin Epidemiol* **44**: 1255–1261

Kono S, Imanishi K, Shinchi K and Yanai F (1993) Serum lipids and left-sided adenomas of the large bowel: an extended study of self-defense officials in Japan. *Cancer Causes Control* **4**: 117–121

Kune GA, Kune S, Read A, Macgowan K, Penfold C and Watson LF (1991) Colorectal polyps, diet, alcohol, and family history of colorectal cancer: a case–control study. *Nutr Cancer* **16**: 25–30

Lee IM, Paffenbarger RS Jr and Hsieh CC (1991) Physical activity and risk of developing colorectal cancer among college alumni. *J Natl Cancer Inst* **83**: 1324–29

Little J, Logan RFA, Hawtin PG, Hardcastle JD and Turner ID (1993) Colorectal adenomas and energy intake, body size and physical activity: a case–control study of subjects participating in the Nottingham faecal occult blood screening programme. *Br J Cancer* **67**: 172–176

Longnecker MP, Gerhardsson DE Verdier M, Frumkin H and Carpenter C (1995) A case–control study of physical activity in relation to risk of cancer of the right colon and rectum in men. *Int J Epidemiol* **24**: 42–50

Longnecker MP, Chen MJ, Probst-Hensch NM, Harper JM, Lee ER, Frankl HD and Haile RW (1996) Alcohol and smoking in relation to the prevalence of adenomatous colorectal polyps detected at sigmoidoscopy. *Epidemiology* **7**: 275–280

Mannes GA, Maier A, Thieme CH, et al (1986) Relation between the frequency of colorectal adenoma and the serum cholesterol level. *N Engl J Med* **315**: 1634–1638

Neugut AI, Johnsen CM and Fink DJ (1986) Serum cholesterol levels in adenomatous polyps and cancer of the colon. *JAMA* **255**: 365–367

Neugut AI, Garbowski GC, Lee WC, et al (1993*a*) Dietary risk factors for the incidence and recurrence of colorectal adenomatous polyps. A case–control study. *Ann Intern Med* **118**: 91–5

Neugut AI, Jacobson JS and Devivo I (1993*b*) Epidemiology of colorectal adenomatous polyps. *Cancer Epidemiol Biomarkers Prev* **2**: 159–176

Pollock AM and Quirke P (1991) Adenoma screening and colorectal cancer (letter). *Br Med J* **303**: 3–4

Potter JD, Slattery ML, Bostick RM and Gapstur SM (1993) Colon cancer: a review of the epidemiology. *Epidemiol Rev* **15**: 499–545

Richardson MT, Leon AS, Jacobs DR JR, Ainsworth BE and Serfass R (1994) Comprehensive evaluation of the Minnesota Leisure Time Physical Activity Questionnaire. *J Clin Epidemiol* **47**: 271–281

Rimm EB, Giovannucci EL, Stampfer MJ, Colditz GA, Litin LB and Willett WC (1992) Reproducibility and validity of an expanded self-administered semiquantitative food frequency questionnaire among male health professionals. *Am J Epidemiol* **135**: 1114–1126

Sandler RS, Lyles CM, Peipins LA, Mcauliffe CA, Woosley JT and Kupper LL (1993) Diet and risk of colorectal adenomas: macronutrients, cholesterol, and fiber. *J Natl Cancer Inst* **5**: 884–891

Sandler RS, Pritchard ML and Bangdiwala SI (1995) Physical activity and the risk of colorectal adenomas. *Epidemiology* **6**: 602–606

Siconolfi SF, Lasater TM, Snow RCK and Carleton RA (1985) Self-reported physical activity compared with maximal oxygen uptake. *Am J Epidemiol* **122**: 101–5

Steinmetz KA and Potter JD (1991) Vegetables, fruit, and cancer. I. Epidemiology. *Cancer Causes Control* **2**: 325–357

Steinmetz KA, Kushi LH, Bostick RM, Folsom AR and Potter JD (1994) Vegetables, fruit, and colon cancer in the Iowa Women's Health Study. *Am J Epidemiol* **139**: 1–15

Stryker SJ, Wolff BG, Culp CE, Libbe SD, Ilstrup DM and Maccarty RL (1987) Natural history of untreated colonic polyps. *Gastroenterology* **93**: 1009–1013

Tornberg SA, Holm LE, Carstensen JM, et al (1986) Risks of cancer of the colon and rectum in relation to serum cholesterol and beta-lipoprotein. *N Engl J Med* **315**: 1629–1633

Washburn RA, Goldfield SRW, Smith KW and Mckinlay JB (1990) The validity of self-reported exercise-induced sweating as a measure of physical activity. *Am J Epidemiol* **132**: 107–113

Willett WC (1990) *Nutritional Epidemiology*. Oxford University Press: New York

© Cancer Research Campaign 1997